# Unmanned Surface Vehicle Collision Avoidance Path Planning in Restricted Waters Using Multi-Objective Optimisation Complying with COLREGs

**DOI:** 10.3390/s22155796

**Published:** 2022-08-03

**Authors:** Yang Gu, Zhenwei Rong, Huzhou Tong, Jia Wang, Yulin Si, Shujie Yang

**Affiliations:** 1 School of Marine Engineering Equipment, Zhejiang Ocean University, Zhoushan 316022, China; yanggu@stu.just.edu.cn (Y.G.); z20095136020@zjou.edu.cn (H.T.); 2 Institute of Ocean Engineering and Technology, Ocean College, Zhejiang University, Zhoushan 316021, China; zw_r@zju.edu.cn (Z.R.); yulinsi@zju.edu.cn (Y.S.); 3 School of Mechanical Engineering, Jiangsu University of Science and Technology, Zhenjiang 212100, China; wjjzhb@just.edu.cn; 4 Hainan Institute of Zhejiang University, Sanya 572025, China

**Keywords:** unmanned surface vehicle, collision avoidance, path planning, multi-objective optimisation, COLREGs, hardware-in-the-loop test

## Abstract

Navigation safety is one of the primary operational requirements for unmanned surface vehicles (USVs) in a complex marine environment, mainly guaranteed by a reliable path planning system for collision avoidance. This work proposes a novel weighted sum multi-objective optimisation strategy for USV collision avoidance path planning in restricted waters. In particular, the coefficients of different objectives could be tuned to emphasise the most critical design consideration under varying navigation scenarios. Moreover, in addition to the Convention on the International Regulations for Preventing Collisions at Sea (COLREGs), the terrain and weather constraints were also considered in the path planning system. The proposed USV collision avoidance path planning framework’s effectiveness was demonstrated through numerical simulations and hardware-in-the-loop (HIL) tests. The numerical simulation results indicate that the proposed method could avoid collision with dynamic and static obstacles, and it is also adaptive to different navigation restrictions and preferences. Moreover, a USV navigation platform was established by incorporating true Automatic Identification System (AIS) signals, and HIL tests were performed with real-time AIS data in a water channel in the Zhoushan archipelago. The results demonstrate that the proposed USV path planning strategy is applicable in restricted waters with complex terrains and weather constraints.

## 1. Introduction

As one kind of autonomous system, unmanned surface vehicles (USVs) need to perceive and interact with the external marine environment to adapt to complex navigation scenarios and ensure safety. Since the USV is usually sailing in an unknown and dynamic marine environment, obstacles may appear in the planned global path at any time. In this case, the USV must find a way to avoid the obstacles but still allow itself to reach the destination and complete the mission successfully [1,2]. Therefore, based on the planned global path, the USV should also be able to perform real-time local obstacle avoidance to meet the requirements of successful and safe navigation [3,4]. Addiitonally, recent reports have indicated that many marine collision accidents are related to human decision failures concerning compliance with the Convention on the International Regulations for Preventing Collisions at Sea (COLREGs) [5,6]. Therefore, it is also necessary to include COLREGs as an integral element in the USV collision avoidance path planning system.

In the past few years, USV collision avoidance path planning has attracted extensive attention from both the academia and industry, and the algorithms can be generally classified into two categories. One is to treat USVs as autonomous mobile robots and use model-driven or data-driven approaches for path planning, such as artificial potential fields [7,8], fuzzy logic [9,10] and the velocity obstacle method [11]. These strategies could be very useful in dynamic situations in open water areas for obstacle avoidance, as USVs are only seen as agile robotic systems without marine navigation restrictions. However, these studies have not incorporated COLREGs into the path planning system, which might cause potential security risks when the USV meets regular marine ships in a complex marine environment. Therefore, the other category is to incorporate COLREGs into the path planning system, which is supposed to keep USVs behaving similarly to other regular crewed ships to avoid collisions. Zhao et al. proposed a COLREG-compliant deep reinforcement learning method for USV collision avoidance [12], but COLREGs was only used to determine the turning direction in the reward. Zaccone et al. proposed an optimal path planning algorithm based on rapidly exploring random trees, which was also compliant with the COLREGs [13]. However, the collision avoidance system did not include weather constraints, etc., which might lead to a nonoptimal solution under complex sea states. Hu et al. developed a multi-objective optimisation framework for path replanning, which was flexible and scalable to accommodate multiple objectives, and mathematical representations of COLREGs and other USV constraints were introduced for the first time [14]. They further improved the multi-objective optimisation framework for path replanning in [15], where a hierarchical sorting rule was designed to prioritise the objective of the course/speed change preference over other objectives. However, the priority of these objectives could have changed in different navigation scenarios, e.g., path smoothness is more important than path distance in a rough sea state and vice versa in energy-saving situations. Additionally, most of these research works were only based on numerical simulations, which were not validated against real-world experiments or hardware-in-the-loop (HIL) tests.

Due to the above-mentioned problems, we propose a novel COLREG-compliant weighted-sum multi-objective optimisation method for USV collision avoidance path planning in restricted waters in this work. In particular, the coefficients of different objective functions could be tuned to emphasise the most critical design consideration in varying navigation scenarios. In addition, the terrain and weather conditions are also considered in USV collision avoidance path planning within restricted water areas. Compared with the existing research studies, the main contributions of this work lie in:

(1) A novel weighted sum multi-objective optimisation framework is established for USV collision avoidance path planning, and the coefficients could be reconfigured for different path preferences.
(2) In addition to conventional COLREGs constraints, the terrain and weather constraints are also considered in USV collision avoidance path planning, which might result in different replanned paths compared with open water areas with calm sea states.
(3) Besides numerical simulations, a novel hardware-in-the-loop USV navigation system is established using an industrial computing platform, and HIL tests in restricted waters with actual Automatic Identification System (AIS) signals and an electronic nautical chart (ENC) are conducted.

The remainder of the paper is organised as follows. Section 2 overviews the USV collision avoidance path planning framework, especially the procedures of the risk assessment and COLREGs rule selection. Section 3 describes how the USV local path replanning problem is transformed into a weighted-sum multi-objective optimisation task and introduces how the COLREG rules and other constraints are mathematically formulated. Section 4 presents the results and analysis of the numerical simulations and HIL tests for various marine encounter situations, particularly under extreme weather and terrain constraints. Section 5 draws the conclusions and points out the limitations of this work and future research directions.

## 2. USV Collision Avoidance Path Planning Framework

**Figure 1:**
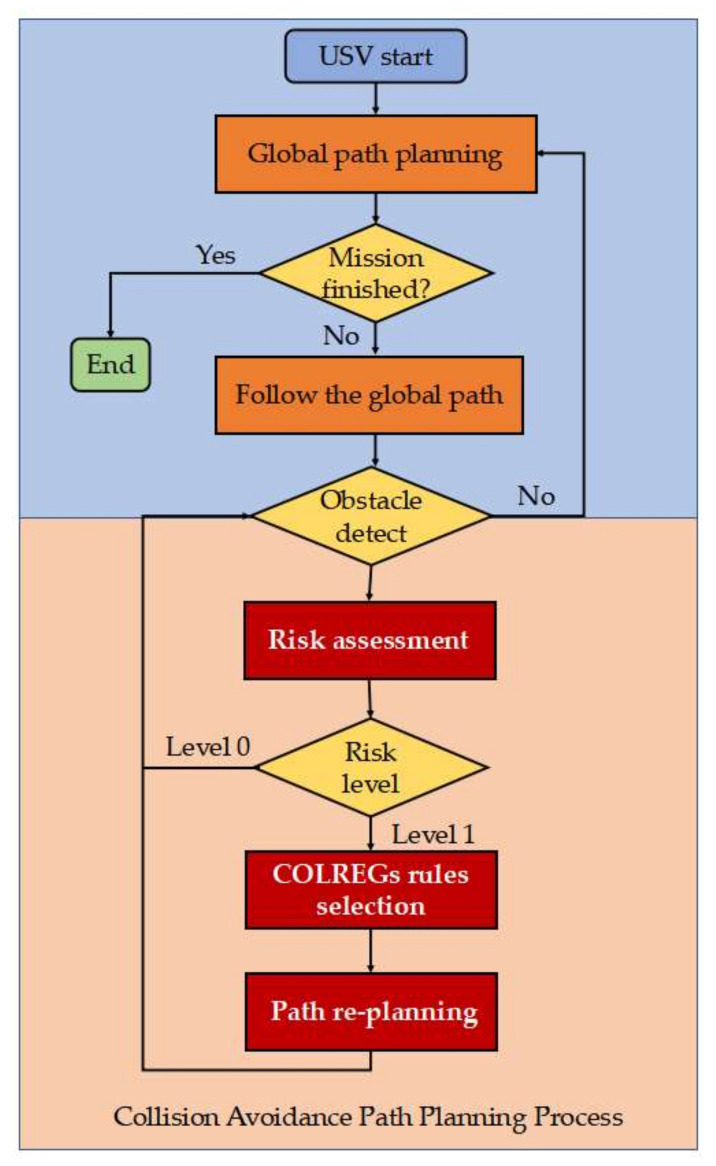
Flowchart of the COLREG-compliant USV collision avoidance path planning process.

The USV collision avoidance path planning system mainly consists of three modules, i.e., risk assessment, COLREGs rule selection and path replanning, which are illustrated in Figure 1. Firstly, risk assessment is performed to reflect the collision probability between the USV and obstacles in nearby waters based on AIS or radar signals. Secondly, based on the encounter situation, the corresponding COLREG rules are selected and used for path replanning, which is critical for USVs to behave in a manner similar to regular-crewed marine ships. At last, path replanning is performed to generate a new local path to avoid collisions with the observed obstacles.

### 2.1. Risk Assessment

**Figure 2:**
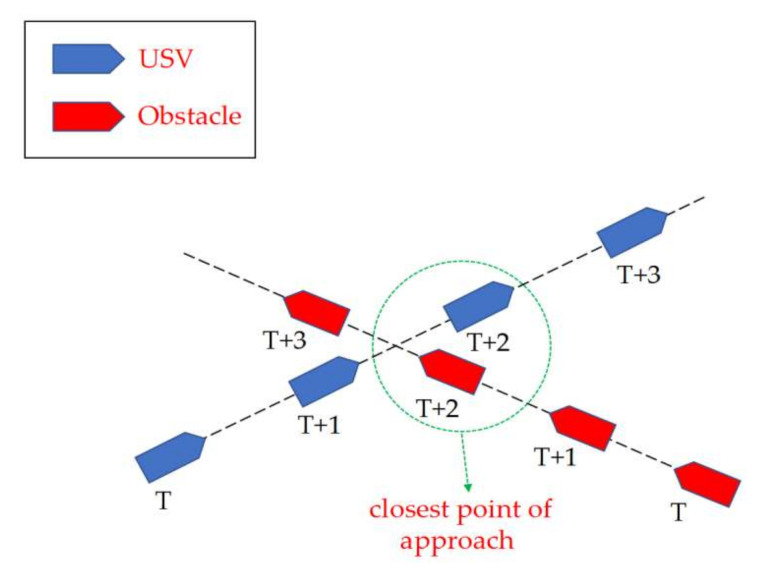
The principle of the closest point of approach.

A reliable risk assessment is the prerequisite for USV collision avoidance, as incorrect assessment results might lead to catastrophic accidents. To assess the risk of collision, the CPA (Closest Point of Approach) method, as depicted in Figure 2, has been widely used in the field of ship obstacle avoidance [16]. This method evaluates whether the USV needs to avoid collision by comparing the time to the closest point of approach (TCPA) and the distance to the closest point of approach (DCPA) with the preset parameters $t_{max}\;$ and $\;d_{max}$ [17]. TCPA $t_{cpa}\;$ and DCPA $d_{cpa}\;$ could be derived with the following formula:

(1) $$t_{cpa}=\left\{\begin{array}{c} 0\;,\;\;\;\;\;\;\;\|v_{A}-v_{B}\|<\varepsilon \;\\ \frac{\left(p_{A}-p_{B}\right)\cdot \left(v_{A}-v_{B}\right)}{\left\|v_{A}-v_{B}\right\|},\;\;\;others \end{array}\right.\;d_{cpa}=\left(p_{A}+t_{cpa}\cdot v_{A}\right)-\left(p_{B}+t_{cpb}\cdot v_{B}\right)\;$$

where $v_{A}$ and $v_{B}$ are the speed of the USV and obstacle, respectively, while $p_{A}$ and $p_{B}$ are their corresponding positions. Additionally, ε  is a preset minimum value.

When the detected obstacle is far enough from the USV, the risk is level 0, which means almost no collision risk. As the relative distance gets closer, the risk level could be changed to level 1 if $t_{cpa}\;$ and $d_{cpa}$ satisfy the following conditions:

(2) $$\left\{\begin{array}{c} 0\leq t_{cpa}\leq t_{min}\\ d_{cpa}\leq d_{min} \end{array}\right.\;\;$$

where $t_{min}=\varepsilon \cdot \mathrm{TCPA}$ and $d_{min}=\varepsilon \cdot \mathrm{DCPA}$ and $t_{min}$ and $\;d_{min}$ represent the minimum collision-free TCPA and DCPA, respectively. Once risk level 1 is confirmed, the USV should select a corresponding COLREG rule and replan the path according to the encounter situation to avoid collisions.

### 2.2. COLREGs Rule Selection

**Figure 3:**
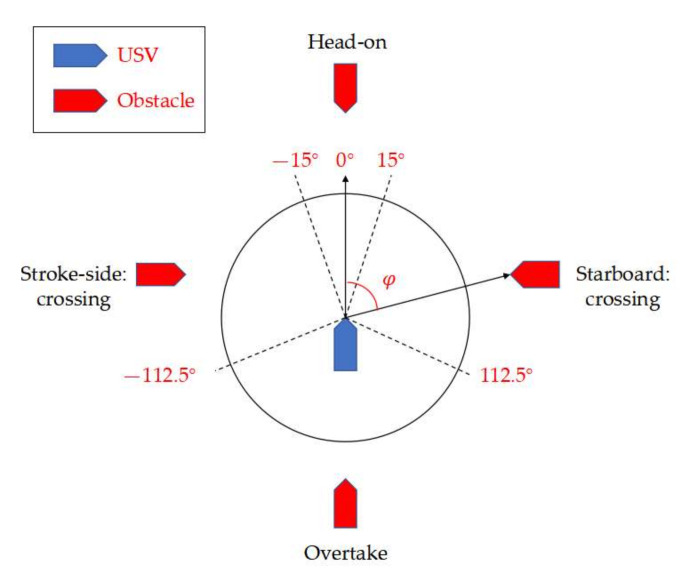
The relative bearing from the obstacle ship to the USV.

In 1977, the International Maritime Organization (IMO) formulated international regulations for preventing collisions at sea for the first time, including the definition and responsibility division of different encounter scenarios and the requirements of acousto-optic early warning in collision avoidance [18]. In general, if a risk of collision appears, the USV needs to judge the encounter situation first to determine the applicable rules and, further, to determine the actions to be taken. According to COLREGs, the encounter situations could be classified into four categories, i.e., head-on, stroke side-crossing, starboard crossing and overtaking. Figure 3 shows the relative bearing from the obstacle ship to the USV, which can be used to determine which situation is encountered. Given the relative bearing of the obstacle ship to the USV, it will then be uniquely determined which situation the USV is facing and the suitable COLREGs rule accordingly.

**Figure 4:**
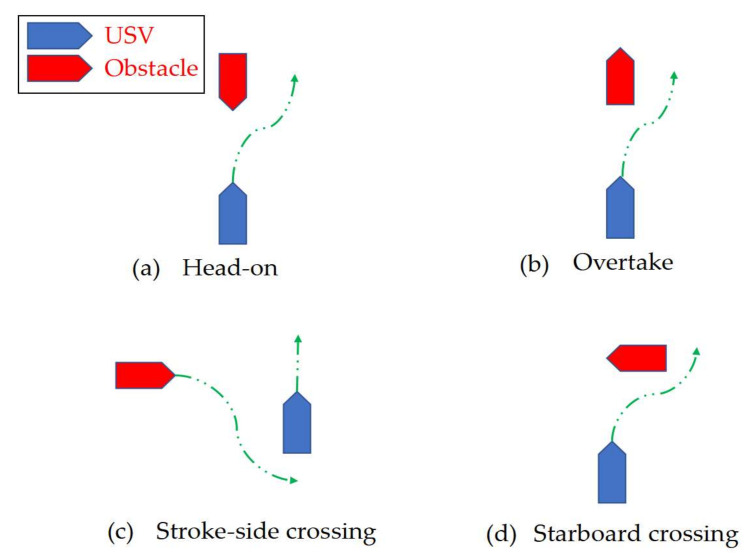
COLREGs rule selection for collision avoidance under different encounter situations.

COLREGs have explicitly defined the rules for avoiding collision in different encounter situations [19]. Firstly, if it is the overtaking case, then any vessel overtaking others should complete the overtaking action from the starboard. Secondly, each head-on ship shall alter its course to starboard, so that each shall pass on the port side of the others in the head-on situation. Moreover, the ship that has others on its starboard side shall keep out of the way in crossing situations. To be more intuitive, these COLREG-related rules are illustrated in Figure 4. It can be noticed that the USV could continue its course only under the stroke side-crossing situation, as shown in Figure 4c, while the USV needs to plan a new path to avoid collision in the other three cases.

### 2.3. Path Replanning

Path replanning is the last and most crucial step of the collision avoidance process. In the complex and dynamic marine environment, path replanning for USVs to avoid collisions should be efficient and reliable. A collision-free path can be planned by generating one or a sequence of sub-waypoints. In this work, single sub-waypoint is used as it is computationally efficient and well-suited for dynamic environments.

**Figure 5:**
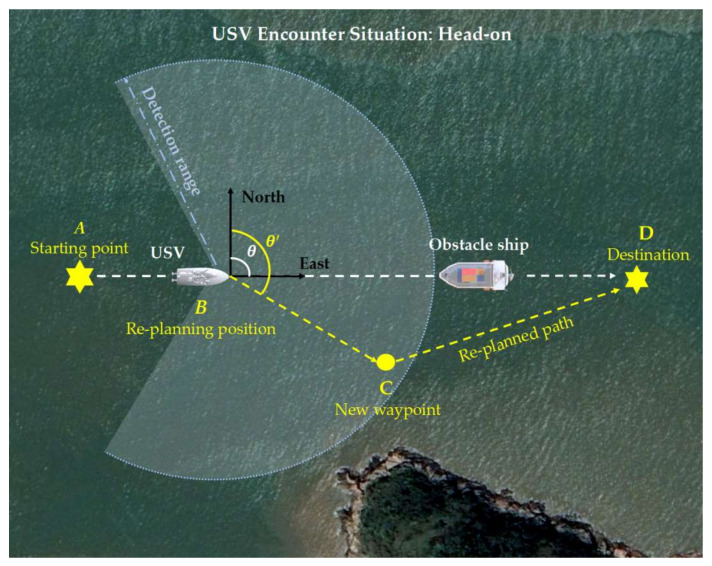
Illustration of a USV collision avoidance path replanning under a head-on situation.

An example of the USV collision avoidance path replanning result is illustrated in Figure 5, which represents a head-on situation. In the beginning, the USV follows the global path from the starting point A to the destination with the heading angle θ. When the obstacle ship appears in the detection range, the USV will keep making risk assessments. If the risk level is elevated to level 1, the appropriate COLREGs rule should be selected, and a new path to avoid collision with the obstacle ship will be planned. Subsequently, the USV will alter its heading angle from θ to θ′ and follow the replanned path to the destination.

In fact, different objectives need to be considered simultaneously when planning the new path for collision avoidance, leading the path replanning itself into a multi-objective optimisation problem. The proposed multi-objective optimisation strategy with COLREGs and other constraints for USV collision avoidance path planning is described in the next section.

## 3. Weighted Sum Multi-Objective Optimisation

A general multi-objective optimisation problem can be represented as follows:

(3) $$\left\{\begin{array}{c} \min\;F\left(x\right)\\ s.t.\;\;\;x\in U \end{array}\right.$$

where *x* is the decision variable vector, *F*(*x*) denotes the objective function and *x∈* U are the relevant constraints. Then, a proper optimisation algorithm could be used to efficiently locate the global optimal solution. Next, we will describe how the USV collision avoidance path planning is achieved using the weighted sum multi-objective optimisation method.

### 3.1. Decision Variables

The decision variable for USV path planning is defined by vector x

(4) $$x:={\left[\begin{array}{cc} \theta^{\prime } & t \end{array}\right]}^{T},$$

where $\theta^{\prime }$ represents the new heading angle of USV to avoid collisions, and t denotes the time required from the replanning position *B* to the new waypoint *C*, as shown in Figure 5. In other words, the replanned path can be determined by the heading angle and travelling time under a specific speed.

### 3.2. Constraints

In fact, COLREGs do not have explicit regulations on navigation constraints for collision avoidance, and in practice, the implementation of COLREGs depends on the understanding of mariners. However, each mariner has a different manner of interpreting these rules, which may result in potential risks and even cause collisions. Therefore, quantitative representations of COLREGs are needed, and the following mathematical inequalities are used to overcome such difficulties, which could also be easily incorporated into the optimisation framework.

1. Safety constraint. Since the highest priority is safety, the first constraint is to eliminate any collision risk, which means represent a risk level of 0.

(5) $$\left\{\begin{array}{c} t_{cpa}\geq t_{min}\\ d_{cpa}\geq d_{min} \end{array}\right.\;$$

2. Heading angle constraint. According to the COLREGs, a heading angle alteration bigger than 15° is large enough to be observed by other ships. It is generally inefficient that the heading angle alteration is bigger than 60°. Therefore, it is assumed that the optimised heading angle variation is between 15° and 60°, i.e.,

(6) $$15{}^{\circ}\leq \left|\theta^{\prime }-\theta \right|\leq 60{}^{\circ}$$

**Figure 6:**
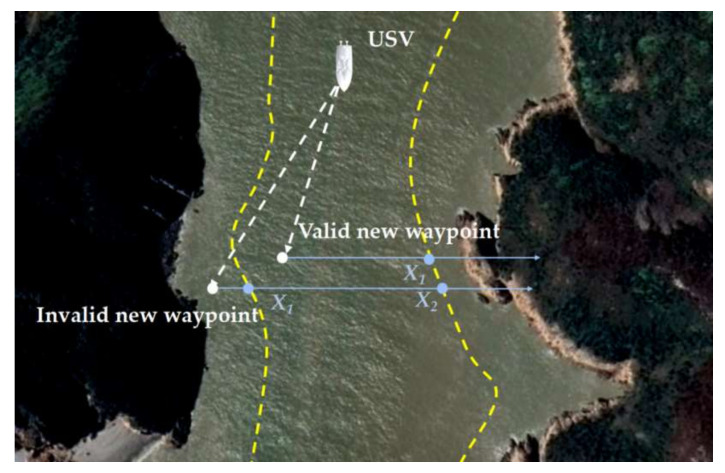
Implementation of terrain constraints using a ray casting algorithm.

3. Time constraint. Once a new path is initiated, the USV continues at least the minimum duration of time to the new waypoint, making the USV’s decision obvious and predictable to other ships. Additionally, the USV should not continue indefinitely on the new path. This is specified by the variable *t*, defining the maximum allowable time constraint,
  (7) $$\underline{t}\leq t\left(x\right)\leq \bar{t},\;$$
  where $\underline{t}$ is the minimum allowable time and $\bar{t}$ is the maximum allowable time. Additionally, terrain and weather constraints are also included, as USVs are prone to conduct collision avoidance actions in restricted waters with terrain constraints, and USVs should avoid large heading angle variations in rough sea conditions.

4. Weather constraint. All the above constraints are considered in mild weather conditions, and certain constraints should be further restricted in rough sea states. For instance, large heading alterations in high sea states are prone to synchronised rolls phenomena, which may pose higher safety risks for USVs [20,21]. Therefore, under high sea states, USV should avoid large heading alterations in the collision avoidance process, and the maximum allowable time could be removed, e.g.,
  (8) $$\left\{\begin{array}{c} 15{}^{\circ}\leq \left|\theta^{\prime }-\theta \right|\leq \varphi_{ss}\\ t\left(x\right)\geq \underline{t} \end{array}\right.\;$$
  where $\varphi_{ss}$ is the maximum allowable heading angle alteration under different sea states.

5. Terrain constraint. Grounding accidents in restricted water areas are the biggest threat to marine navigation safety, which is usually caused by the misinterpretation of the water depth. Additionally, the submerged reef around the coast causes a potential risk during navigation. The terrain constraint could be mainly divided into the following four cases: (1) an area with shallow water depth or irregular water depth variation, (2) long continuous reef veins in their vicinity, (3) the narrow area between reefs and islands without accurate measurements and (4) the isolated reefs and the point beach around shallower beaches. Therefore, the designed USV collision avoidance new waypoint should be within the terrain-permitted region. Mathematically, this could be judged using the ray casting algorithm [22], as shown in Figure 6, where *X_k_* denotes the intersection points between a ray starting from the planned new waypoint and the water area border with an acceptable water depth. If the waypoint is inside of the region, the ray will intersect the border an odd number of times, which means
  (9) max(k) ∈2n+1
  where *n =* 0, 1, 2, …. Then, the constraint could be implemented by simply counting the crossing number.

### 3.3. Objective Functions

As mentioned above, different objectives, such as safety, efficiency, distance and smoothness, need to be considered simultaneously in USV collision avoidance path planning [23,24,25]. Therefore, the following three objectives are considered in the optimisation framework.

1. Safety. The safety objective is the first and foremost one, as it is the primary concern for all marine ships. In other words, this objective is to eliminate all collision risks. Mathematically, this safety objective function could be written as follows [26]:
  (10) $$f\left(x\right)=\left\{\begin{array}{c} d_{min}-DCPA\left(x\right),\;\;\mathrm{if}\;DCPA\left(x\right)\geq d_{min}\\ e^{a\left(d_{min}-DCPA\left(x\right)\right)/TCPA\left(x\right)}-1,\;\mathrm{otherwise}\;\;\; \end{array}\right.$$
  Here, *a* is a constant scaling parameter, and $f\left(x\right)$ represents the deviation between *x*-induced DCPA and minimum collision-free DCPA, which means that there will be no risk of collision only when $f\left(x\right)\leq \;$0. The smaller $\;f\left(x\right)\;$ is, the safer the replanned USV path is.
2. Smoothness. The abrupt change of the heading angle will lead to a potential risk for ships, so the replanned path should be as smooth as possible. The objective is to minimise the sum of angle changes from replanning position *B* to the destination *D*, which is equal to the sum of the angles ∠*B* and ∠*D*. Therefore, ∠*B* + ∠*D* is used to quantify the path smoothness, and it could be derived from basic trigonometry.
  (11) $$g\left(x\right)=\left(\theta^{\prime }-\theta \right)+arctan\frac{vtsin\left(\theta^{\prime }-\theta \right)}{l_{BD}-vtcos\left(\theta^{\prime }-\theta \right)}\;,\;$$
  where $l_{BD}$ is the shortest distance from the replanning point to the destination, and v is the USV speed.
3. Distance. The replanned path should be as short as possible to reduce energy consumption. This objective function will minimise the deviation between the changed path and the original path.
  (12) $$h\left(x\right)=vt+l_{CD},\;$$
  where $l_{CD}$ is the distance from the new waypoint *C* to destination *D*.

In general, there are mainly two methods to solve multi-objective optimisation problems, i.e., Pareto and scalarisation [27]. The Pareto method treats each objective as equal weight, and it could generate the desired solution if there is a tradeoff among different objectives. However, the preference for USV path planning might change with weather conditions and other marine conditions, so the “equal objective” approach may not always be suitable. In addition, the roughness of the objective function is easy to result in the local optimal solution [15]. Therefore, the scalarisation method is used in this work, which could create multi-objective functions into a single solution using weights. Moreover, the weighted sum optimisation method could achieve the path preference results under varying navigation occasions by tuning the weight coefficients. As a result, the path replanning multi-objective optimisation problem could be rewritten as

(13) $$\left\{\begin{array}{c} min\;\;w_{f}f\left(x\right)+w_{g}g\left(x\right)+w_{h}h\left(x\right)\\ s.t.\;\left(5\right)-\left(9\right)\; \end{array}\right.,$$

where $w_{f}$, $w_{g}$ and $w_{h}$ are the weights of the safety, smoothness and distance objectives, respectively.

### 3.4. Optimisation Algorithm

**Figure 7:**
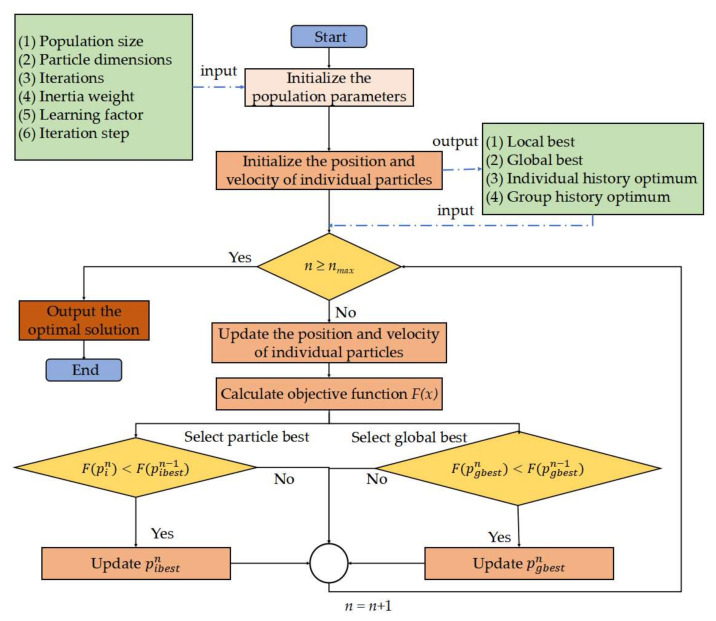
Workflow of the CPSO algorithm.

The particle swarm optimisation (PSO) algorithm has been widely used to find the optimal solution for optimisation problems due to its simplicity and low parameterisation characteristics. In order to further improve the convergence rate, constricted particle swarm optimisation (CPSO) is used in this work to find the optimal solution for USV path replanning [28], where a constriction factor is introduced into the velocity rule, which helps reduce the particle velocities thereby ensures convergence. The workflow of the CPSO algorithm is illustrated in Figure 7, which could be mainly divided into the following three steps.

1. Initialise the population: The population P  is a set of n particles, each with its position and velocity. For every particle in the population, its position is randomly initialised in the decision space, and its velocity is initially set to 0, and the archive is initialised as an empty set.
2. Select the local and global best: The local best $p_{ibest}^{n}$ is the best position of the particle achieved, while the global best $\;p_{gbest\;}^{n}\;$ is the best position in the population, which are all selected according to the proposed objective function.
3. Update the position and velocity of individual particles: The population of the particles moves in the search space according to two simple mathematical formulae for the particle’s position and velocity as follows:

(14) $$\left\{\begin{array}{c} v_{i}\left(k\right)={\lambda v}_{i}\left(k-1\right)+c_{1}r_{1}\left({\mathrm{Pb}}_{i}\left(k-1\right)-x_{i}\left(k-1\right)\right)+c_{2}r_{1}\left(Gb_{j}\left(k-1\right)-x_{i}\left(k-1\right)\right)\\ x_{i}\left(k\right)=x_{i}\left(k-1\right)+v_{i}\left(k\right)\\ \begin{array}{c} \lambda =2/\left|2-\varphi -\sqrt{\left(\varphi^{2}-4\varphi \right)}\right|\\ \varphi =c_{1}+c_{2} \end{array} \end{array}\right.$$

where $x_{i}\left(k\right)={\left[x_{i1}^{T}\left(k\right),\;\ldots ,x_{in}^{T}\left(k\right)\right]}^{T}\;,$ $x_{i}\left(k\right)$ is the position of the ith particle at the kth iteration, and $x_{i}\left(k\right)\in \left[x_{min},\;x_{max}\right]$ with $x_{min}$ and $x_{max}$ are the lower and upper bounds for all particles’ positions. $v_{i}\left(k\right)={\left[v_{i1}^{T}\left(k\right),\ldots \;,v_{in}^{T}\left(k\right)\right]}^{T}$, where $v_{i}\left(k\right)$ is the velocity of the *i*th particle at the *k*th iteration. ω is the inertia weight, and c1 and c2 are called acceleration coefficients, namely, cognitive and social parameters, respectively. λ is the constriction factor, which can govern the convergence of the multi-objective optimisation of USV path planning with inequality constraints. r1 and r2 are two uniform random number samples from [0, 1]. $Pb_{i}\left(k\right)$ is the local best position encountered by the *i*th particle at the *k*th iteration, and $Gb_{j}\left(k\right)$ is the *j*th particle in the current archive.

Note that different from Pareto multi-objective optimisation where the roughness of the Pareto objective functions is easy to result in a local optimal solution, the weighted sum optimisation method has smoother objective functions, which is greatly helping prevent the optimisation algorithm from finding the local optimal solutions. In addition, the population size and the number of iterations should be increased to reduce the probability of falling into the local optimum.

## 4. Numerical Simulations and Hardware-in-the-Loop Tests

In order to evaluate the performance of the proposed USV collision avoidance path planning method, both numerical simulations and hardware-in-the-loop (HIL) tests were performed. Numerical simulations were executed in the Matlab 2016a environment running on a workstation with a 32-core i7 processor and 64 GB RAM, and the CPSO algorithm is set with a population of 40 particles with a maximum of 150 generations for all the simulations. Furthermore, the HIL tests are performed on the established USV navigation platform at Zhejiang Ocean University, and the USV collision avoidance path planning is performed in restricted water areas using the obtained true AIS signals and electronic nautical chart.

Since the weighted sum method is used in this work, proper scalings or normalisation of the objectives are needed so that the ranges or values of each objective should be comparable. Therefore, we firstly derive the Pareto multi-objective optimisation solutions, since they do not rely on weight coefficients, and the reference values for *f(x)*, *g(x)* and *h(x)* are obtained as −1.738, 20.097, and 1.505, respectively. In order to ensure that the three objectives have the same proportion in the weighted sum, the normalisation process is needed so that |*w_f_* *f(x)*| = |*w_g_* *g(x)*| = |*w_h_* *h(x)*| and $\sum_{i=0}^{n}w_{i}=1,\;w_{i}\in \left(0\;,\;1\right)$. Therefore, the reference weight coefficients could be obtained as wf=0.45 and wg=0.04, while wh=0.51.

In this work, the following two scenarios are considered in the weight coefficient tuning process. Additionally, note that the safety objective has the same weight under different scenarios, since it is the most important design factor for USV collision avoidance path planning. (1) Energy-Saving Scenario: In order to save energy consumption with the collision avoidance resulted longer routes, it is necessary to reduce the additional cruising distance as much as possible, so that the weight coefficient *wh* of distance objective function is increased to 0.53 in this case. (2) Rough Sea Scenario: When facing severe weather conditions, it is suggested that the USV should avoid big heading angle alteration to improve the cruising safety. Therefore, the smoothness objective weight coefficient *wg* should be tuned under different sea states. As shown in the Table 1, *wg* is elevated (represented by ↑) from 0.04 to 0.08, 0.10 and 0.12 under sea state levels 3–5, respectively, while *wh* is decreased (denoted by ↓) to 0.47, 0.45, 0.43 from 0.51. More severe sea states are not considered here, as normally, marine ships will be prohibited from cruising when the sea state exceeds level 5.

Additionally, the following $\varphi_{ss}$ settings in Table 2 are used to constrain the heading angle alteration in the replanned path under different rough sea conditions, which means when the sea state reaches Level 3, reduced $\varphi_{ss}$ will be used in the path planning optimisation process.

### 4.1. Numerical Simulations

**Figure 8:**
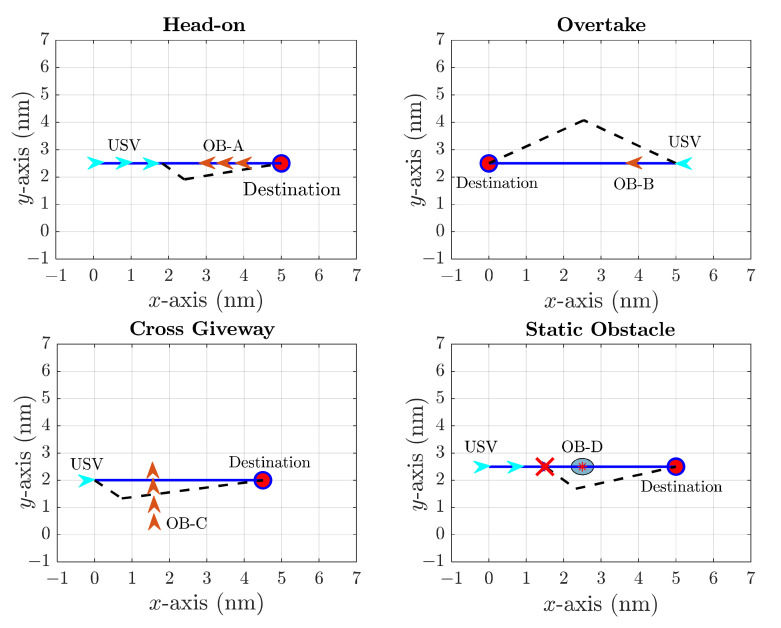
Numerical simulation results for USV path planning with dynamic and static obstacles.

The numerical simulation results involving both dynamic and static obstacle collision avoidance are shown in Figure 8, where OB-A, OB-B and OB-C are the dynamic obstacle ships, and OB-D is a static obstacle. The light blue arrow represents the USV at different time point, while the dark red arrow stands for the obstacle ship. The blue line represents the original path, while the black dash line denotes the collision-free path. As shown in Figure 8, the dynamic situations, including head-on, Overtake and Cross Giveway situations, are defined by COLREGs. It is clearly shown that the USV could not only find a collision-free path in dynamic situations but also bypasses the static obstacles.

**Figure 9:**
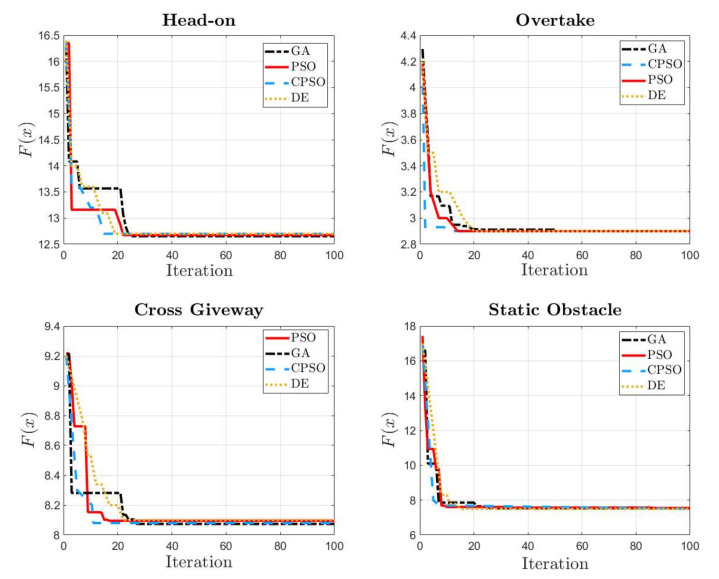
Convergence comparison of PSO, CPSO, GA and DE.

In order to better demonstrate the performances of CPSO with other algorithms, the genetic algorithm (GA) and differential evolutionary (DE) methods are also used for comparison. It can be seen from the Figure 9 that, compared with the standard PSO, GA and DE, CPSO leads to faster convergence for USV path planning optimisation due to the introduction of the constricted factor.

**Figure 10:**
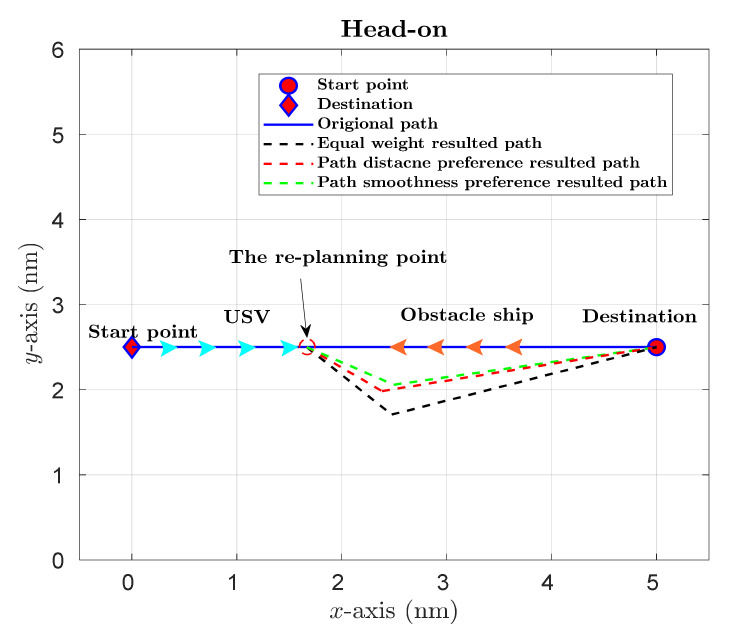
Path planning results with different weight coefficients under a head-on situation.

**Figure 11:**
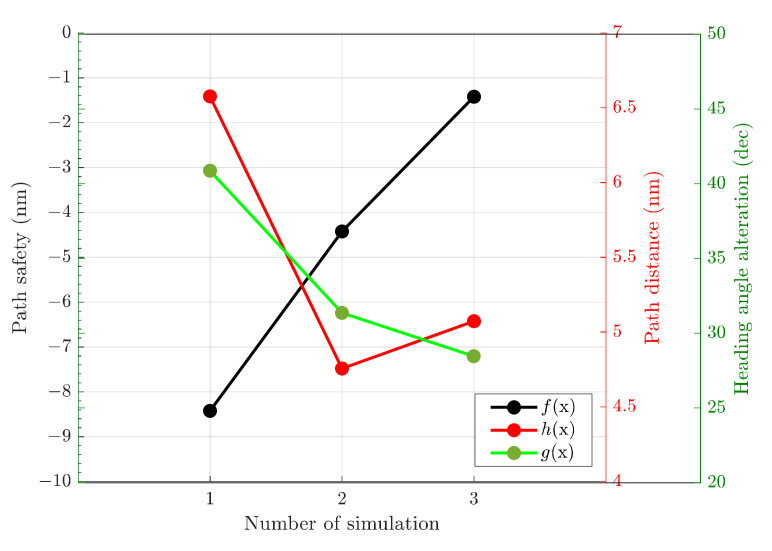
Objective comparison with different weight coefficients ((1) equal weight, (2) path preference and (3) smoothness preference).

Additionally, one of the main advantages for the proposed weighted sum multi-objective optimisation strategy is that the coefficients could be adjusted for different path planning preferences. For instance, the distance objective $h\left(x\right)$ should be equipped with a considerable weight if energy-saving is the primary concern in path planning. To better demonstrate this feature, numerical simulations with different weight coefficients have also been performed, and the results are illustrated in Figure 10 and Figure 11. It could be observed from Figure 10 that (1) the black line represents the collision avoidance path with equal weights, which has the most significant heading angle alteration and the longest path distance, while the safety objective has the best result, (2) compared to equal weights, the result with a distance preference (red dash line) has a smaller heading angle alteration and a shorter path distance, and (3) the green dash line shows path smoothness, a preference-induced result, which has the smallest heading angle alteration, but the safety objective is the worst among the three solutions. The above results indicate that the proposed strategy not only manages to result in a collision avoidance path with different preferences but also shows that the three objectives are contradictory with each other, and there are no paths that minimise all three objectives simultaneously. Quantitative comparisons of different objectives regarding safety, smoothness and distance are shown in Figure 11, which clearly illustrates the influences of weight coefficients on the optimisation results.

### 4.2. Hardware-in-the-Loop Tests

**Figure 12:**
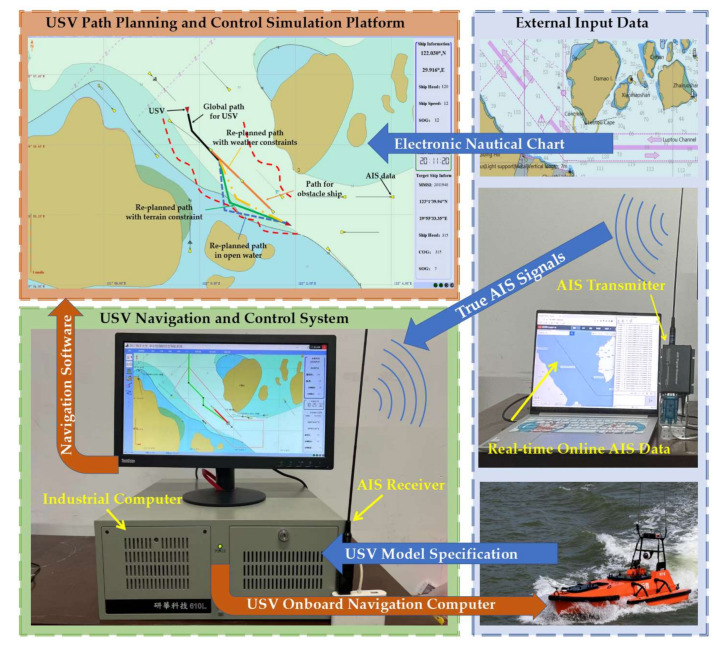
USV collision avoidance path planning HIL test platform.

The proposed USV collision avoidance path planning strategy has been preliminarily evaluated with numerical simulations. To further validate its effectiveness, HIL tests are conducted using the established USV navigation platform at Zhejiang Ocean University, which is illustrated in Figure 12.

The HIL platform consists of (1) an industrial computer that is identical to the USV shipborne navigation computing platform; (2) an AIS receiver, acting similarly to the USV AIS system by receiving online real-time AIS data through a wireless transmitter and (3) an USV path planning and control simulation environment, in which USV collision avoidance path planning and path tracking control simulations could be performed. Note that the introduction of AIS signals and electronic nautical charts, as well as USV model specification and motion dynamics, form a convenient and unique measure to test the USV navigation system in a safe “real-world” environment. Moreover, compared with numerical simulations, nautical charts and true AIS data are used in the HIL tests so that the navigation computer and software can be easily migrated into USVs if the tests work well.

**Figure 13:**
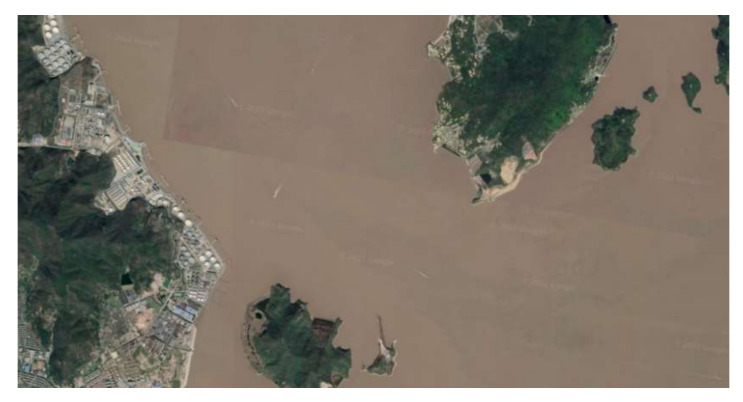
Luotou Channel in the Zhoushan Archipelago (Google Earth 29°55′19.99″ N and 122°05′07.00″ E).

**Figure 14:**
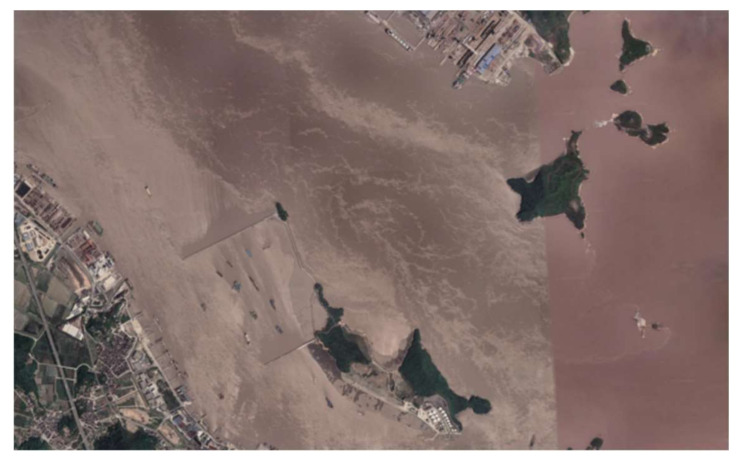
The water area at Zhoushan Xiushan Island (Google Earth 30°08.00″ N and 122°08.00″ E).

Collision avoidance in the real-world marine environment often occurs in restricted waters, and it is particularly challenging when it occurs in water channels. In this work, in order to demonstrate the effectiveness of the proposed approach in restricted water areas, the Luotou Channel (29°55′19.99″ N and 122°05′07.00″ E) in the Zhoushan Archipelago and the water area (30°08.00″ N and 122°08.00″ E) around Zhoushan Xiushan Island are selected for the HIL tests, and the target water areas as shown in Figure 13 and Figure 14 are used for the USV collision avoidance HIL tests. The initial position and global path for USV are imported into the electronic nautical chart for collision avoidance path planning.

**Figure 15:**
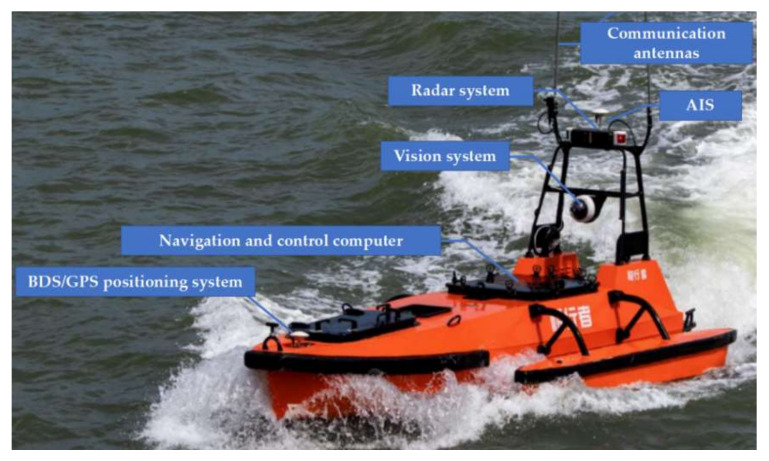
“M80 Polar Walker” USV developed by Yunzhou-Tech Ltd. [29].

To better demonstrate the USV collision avoidance behaviours, USV motion dynamics and path reference tracking control are also incorporated into the HIL simulation environment. In this work, the “M80” USV with rudderless double thrusters developed by Yunzhou-Tech Ltd, Zhuhai, China. is used as the USV model [29]. As shown in Figure 15, the AIS, radar, GPS, camera and communication system are installed on the USV, and its parameters are listed in Table 3.

**Figure 16:**
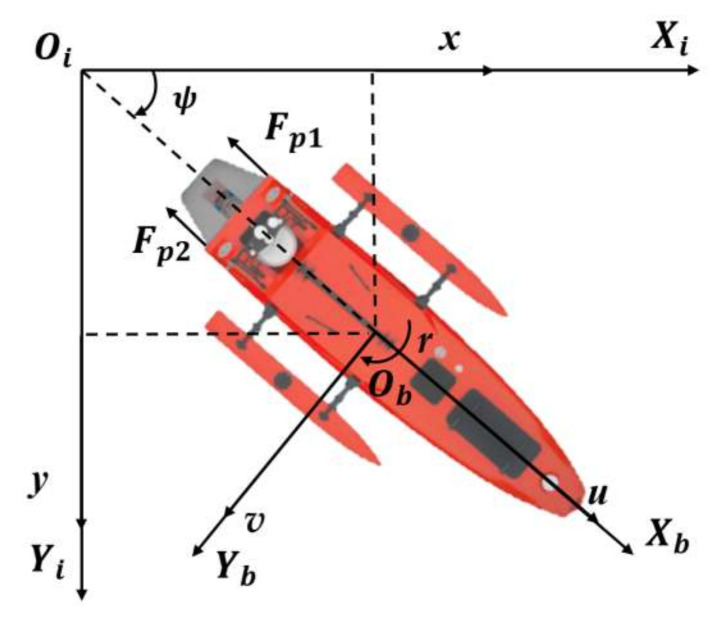
The motion coordinates of “M80” USV.

In order to determine the USV motion equations, two coordinates are used as depicted in Figure 16, i.e., inertial frame *O_i_X_i_Y_i_* and body frame *O_b_X_b_Y_b_*. Then, the 3-DOF motion equation could be established as follows [30]:

(15) $$\left\{\begin{array}{c} \left(m+m_{\dot{u}}\right)\dot{u}-\left(m+m_{\dot{v}}\right)rv+D_{u}u=F_{p1}+F_{p2}\\ \left(m+m_{\dot{v}}\right)\dot{v}+\left(m+m_{\dot{u}}\right)ru+D_{v}v=0\\ \left(I_{z}+I_{\dot{r}}\right)\dot{r}-\left(\left(m+m_{\dot{u}}\right)-\left(m+m_{\dot{v}}\right)\right)vu+D_{r}r=\left(F_{p1}-F_{p2}\right)d_{p}\\ \begin{array}{c} F_{p1}=\left(1-t_{p}\right)\rho D^{4}K_{T}\left(J_{0}\right)n_{1}\left|n_{1}\right|\\ F_{p2}=\left(1-t_{p}\right)\rho D^{4}K_{T}\left(J_{0}\right)n_{2}\left|n_{2}\right| \end{array} \end{array}\right.$$

where u and *v* are the USV surge and sway speed, and r  is the yaw angular velocity. m is the mass of USV, and $m_{\dot{u}}\;$ and $\;m_{\dot{v}}\;$ represent the added mass along surge and sway directions in the body frame, respectively. $I_{z}\;$ is the yaw moment of inertia, and $J_{\dot{r}}\;$ is the corresponding added inertia. $D_{u},\;D_{v}$ and $D_{r}$ are the drag coefficients for the USV surge, sway and yaw motions, respectively. $F_{p1}$ and $F_{p2}\;$ denote the thrust forces from the two thrusters, while *n_1_* and *n_2_* are their rotational speeds. $d_{p}\;$ is the transverse distance from the USV centreline to each thruster, ρ  is the water density, *D* is the diameter of the propeller, $t_{p}$ represents the thrust reduction coefficient and $K_{T}$ is a thrust coefficient, depending on the advanced ratio $J_{0}$. Additionally, proportional integral derivative (PID) control is used here for USV reference path tracking [31,32,33].

**Figure 17:**
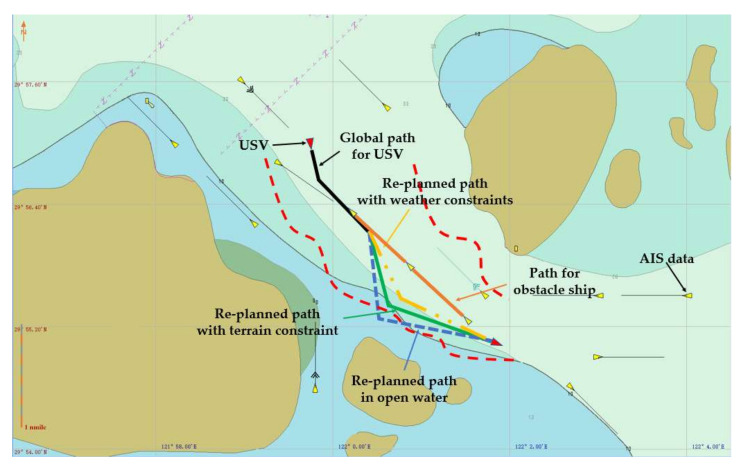
HIL test results for for a representative head-on encounter in restricted water.

**Figure 18:**
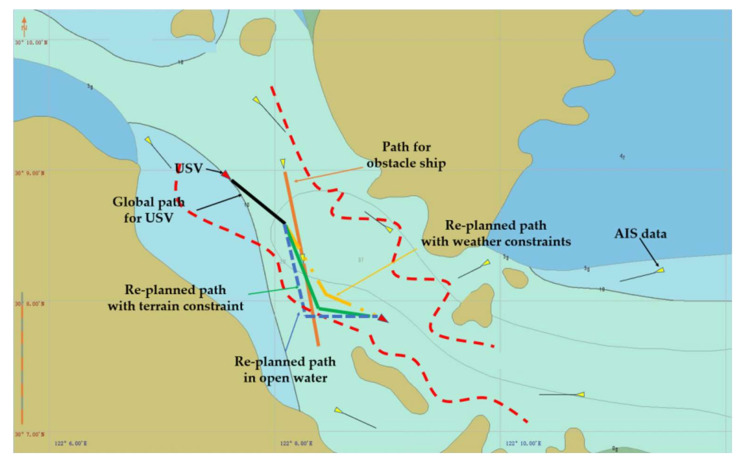
HIL test results for for a representative overtaking encounter in restricted water.

The USV collision avoidance HIL test results for the representative Head-on and Overtaking encounter situations are shown in Figure 17 and Figure 18, respectively, where the USV should give way to another ship on its starboard side according to COLREGs. For Head-on encounter situation, USV navigates the restricted waters along the planned global path (black line). It is then detected that there is an obstacle ship heading towards the USV (the orange line). When USV sees a risk of collision, a new path could be planned (green line) based on the proposed strategy under terrain constraints, and the USV will change its heading angle to avoid collision and follow the replanned path. Under the overtaking encounter situation, USV also manages to detect the obstacle ship and creates a new path to avoid collision.

Furthermore, in order to comparatively evaluate the influences of terrain and weather constraints, HIL tests in open water and severe weather conditions are also conducted. As seen in Figure 17 and Figure 18, compared to the replanned path in restricted water areas (represented by red dash curves), the generated path in open water (blue dotted line) will exceed the terrain limits. Additionally, the path planning results for rough sea conditions (yellow dotted line) will lead to a smaller heading angle alteration. Therefore, it is demonstrated that the proposed USV path planning strategy could work in restricted waters with complex terrain and weather constraints.

**Figure 19:**
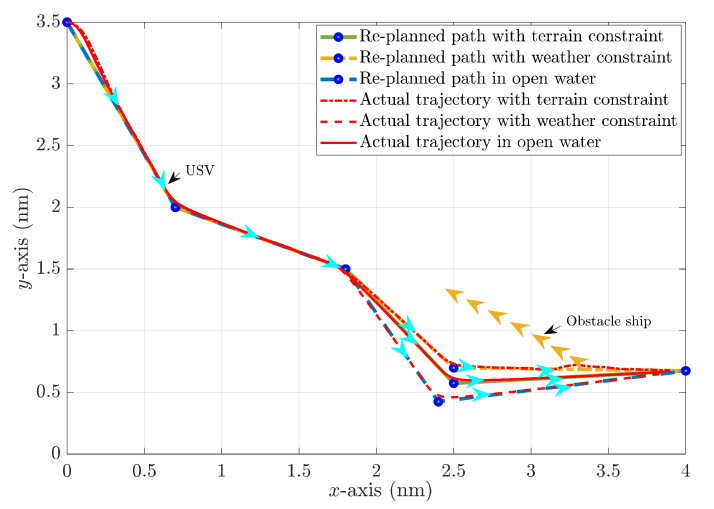
Collision avoidance paths and actual trajectories of USV in a head-on encounter situation with different constraints.

**Figure 20:**
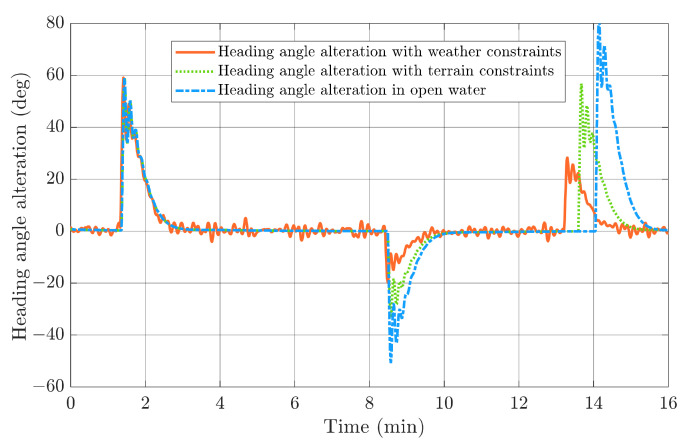
Heading angle alterations with different constraints in head-on encounter situations.

The HIL test results in a head-on encounter situation regarding the replanned paths and actual USV trajectories with different constraints plotted in Figure 19, and the heading angle alternations are shown in Figure 20. It can be clearly noticed that USV will have the longest replanned path and the most significant heading angle alternation without weather and terrain constraints, and the heading angle variation could even reach 80°. In comparison, USV could find a shorter and safer path under the proposed multi-objective optimisation with terrain constraint. Additionally, notice that the heading angle under rough sea states is not as stable as that in a calm sea, so the USV should avoid large heading angle alternations during navigation. This corresponds well to the path planning result under weather constraints, where the maximum heading angle variation is around 30°.

**Figure 21:**
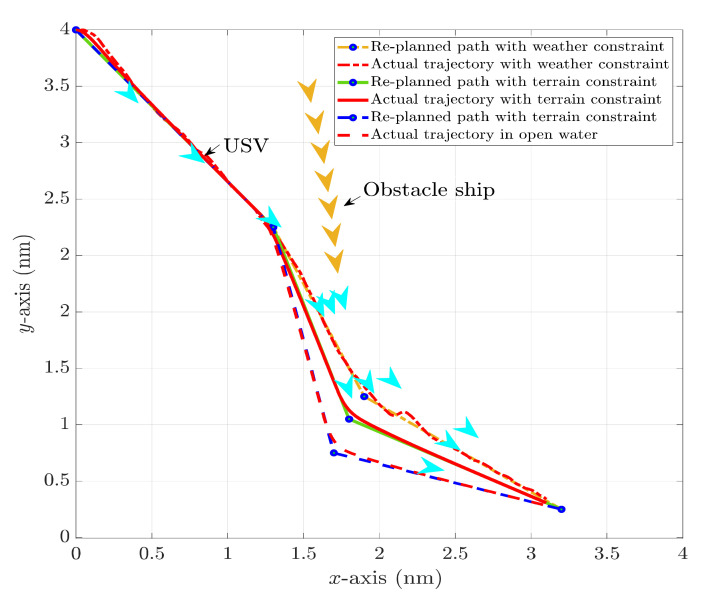
Collision avoidance paths and actual trajectories of USV under overtaking encounter situations with different constraints.

**Figure 22:**
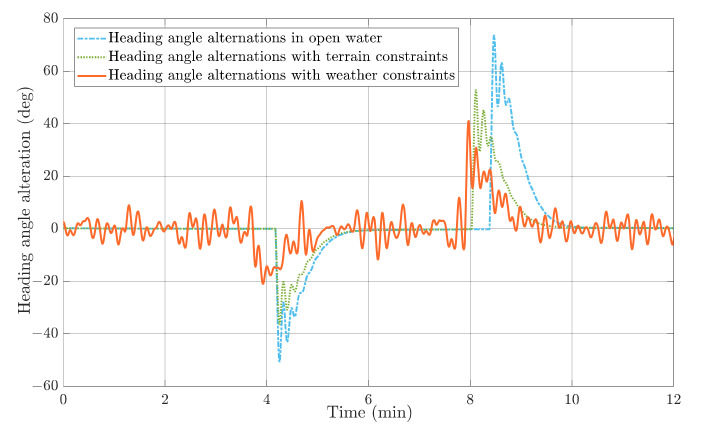
Heading angle alterations with different constraints under overtaking encounter situations.

The HIL test results in overtaking the encounter situation regarding the replanned paths and actual USV trajectories with different constraints are plotted in Figure 21, and the heading angle alternations are shown in Figure 22. It can be again noticed that USV will have the longest replanned path and the most significant heading angle alternation without weather and terrain constraints. In comparison, USV could find a shorter and safer path under the proposed multi-objective optimisation with a terrain constraint.

## 5. Conclusions

In this work, a COLREG-compliant weighted sum multi-objective optimisation method for USV collision avoidance path planning is proposed. In particular, the coefficients of different objective functions could be tuned to emphasise the most critical design consideration in varying navigation scenarios. In addition, the terrain and weather conditions are also considered in USV collision avoidance path planning within restricted water areas. The effectiveness of the proposed collision avoidance path planning scheme has been demonstrated through both numerical simulations and hardware-in-the-loop tests. Numerical simulation results indicate that the proposed method could avoid collision with both dynamic and static obstacles and adapt to different navigation needs. HIL tests are performed on an established USV navigation platform with real-time true AIS signals, and the HIL test results also show that the proposed USV path planning strategy could meet the water area and the heading angle needs in restricted waters with complex terrain and weather constraints. Though COLREGs and other constraints are considered in this work, the formulated USV collision avoidance problem is still based on several assumptions. For instance, the obstacle ship is supposed to strictly obey COLREGs, which might not be feasible in real-world scenarios. These complex situations will be further investigated in future research studies.

## Figures and Tables

**Table 1:** Weight tuning under different sea states.

Sea State	*wf*	*wg*	*wh*
Level:0, Wave height: 0 m, Wind: 0	0.45	0.04	0.51
Level:1, Wave height:0–0.1 m, Wind: 1	0.45	0.04	0.51
Level:2, Wave height:0.1–0.5 m, Wind: 2	0.45	0.04	0.51
Level:3, Wave height:0.5–1.25 m, Wind: 3–4	0.45	0.08↑	0.47↓
Level:4, Wave height:1.25–2.5 m, Wind: 5	0.45	0.10↑	0.45↓
Level:5, Wave height:2.5–4 m, Wind: 6	0.45	0.12↑	0.43↓

**Table 2:** Weather constraints under different sea states.

Sea State	$\varphi_{ss}$
Level:0, Wave height: 0 m, Wind: 0	60°
Level:1, Wave height:0–0.1 m, Wind: 1	60°
Level:2, Wave height:0.1–0.5 m, Wind: 2	60°
Level:3, Wave height:0.5–1.25 m, Wind: 3–4	50°
Level:4, Wave height:1.25–2.5 m, Wind: 5	40°
Level:5, Wave height:2.5–4 m, Wind: 6	30°

**Table 3:** Parameters of the “M80” USV.

Parameters	Vaule
Total length	5.5 m
Breadth	2.4 m
Draft	0.45 m
Maximum speed	10 kn
Mass	1429 kg
Distance between two propellers	0.72 m
Diameter of the propeller	0.3 m

## Data Availability

Not applicable.
